# Emergence of Classical Random Walk from Non-Hermitian Effects in Quantum Kicked Rotor

**DOI:** 10.3390/e27030288

**Published:** 2025-03-10

**Authors:** Wenxuan Song, Jiaming Zhang, Lihao Hua, Zhihua Xiong, Wenlei Zhao

**Affiliations:** School of Science, Jiangxi University of Science and Technology, Ganzhou 341000, China

**Keywords:** quantum walk, non-Hermitian kicked rotor, quantum entanglement

## Abstract

We investigate the quantum random walk in momentum space of a spinor kicked rotor with a non-Hermitian kicking potential. We find that the variance in momentum distributions transitions from quadratic to linear growth over time for the non-Hermitian case. Correspondingly, the momentum distributions are in the shape of Gaussian wavepackets, providing clear evidence of a classical random walk induced by the non-Hermitian-driven potential. Remarkably, the rate of the linear growth of the variance diverges as the non-Hermitian parameter approaches zero. In the Hermitian case, deviations from the quantum resonance condition dramatically suppress the quadratic growth of the variance, leading to dynamical localization of the quantum walk. Under such quantum non-resonance conditions, the classical random walk is significantly reduced by the non-Hermitian-driven potential. Interestingly, non-Hermiticity enhances quantum entanglement between internal degrees of freedom, while deviations from the quantum resonance condition reduce it. Possible applications of our findings are discussed.

## 1. Introduction

Quantum random walks (QRWs) exhibit physics fundamentally distinct from classical random walks (CRWs) due to their unique features, such as quantum superposition and entanglement [1,2,3,4,5]. The intrinsic entanglement between position and internal degrees of freedom enables exponential speedups in quantum algorithms for tasks like database searching and graph analysis [6], providing a theoretical foundation for the applications of QRWs in quantum computation and quantum information science [7,8,9]. Moreover, the QRW model provides an ideal platform for exploring nontrivial one-dimensional topological phases [10,11,12], where edge states, protected by the system’s symmetries, demonstrate robustness against perturbations [13]. By incorporating a periodically driven potential into the QRWs model, one even observes Floquet topological phase transitions, opening an opportunity for exploring fundamental quantum phenomena [14]. Interestingly, QRWs have been adopted as a novel framework, functioning as a unitary map to predict the long-term dynamics of quantum diffusion and reveal the universality of subdiffusion in nonlinear systems [15]. Even more remarkably, experimental advancements in electric circuits [16,17] and ultracold atoms [18,19] have enabled the observation of the rich behaviors exhibited by QRWs, providing direct validation of theoretical predictions.

Non-Hermitian quantum mechanics has been accepted as a theoretical framework for understanding open quantum systems and dissipative phenomena, offering unique insights into fundamental physics and potential applications [20]. The emergence of spontaneous PT symmetry breaking at exceptional points, characterized by the coalescence of eigenstates and eigenvalues, has been acknowledged as an essential modification to traditional quantum mechanics [21,22]. New types of topological invariants, such as winding numbers in complex energy planes, extend traditional classifications of topological phase transitions [23,24,25,26,27], thereby broadening our understanding of conventional bulk–boundary correspondence and non-Bloch band theory [28,29,30]. The coexistence of non-Hermiticity and nonlinearity even induces super-exponential increases in both out-of-time order correlators [31] and mean energy [32], which has significant implications in quantum scrambling in chaotic systems. The transition from the unbroken to broken PT symmetry phase induces the localization of the quantum state and the emergence of edge states for the spatial degrees of freedom in non-Hermitian QRW models [33,34]. Interestingly, advances in photonic lattices have facilitated experimental observations of non-Hermitian edge bursts and self-acceleration in QRWs, paving the way for studying the rich dynamics of non-Hermitian systems [35,36,37,38].

In this context, we investigate the quantum walk dynamics in the momentum space of the spinor kicked rotor model, focusing on non-Hermitian effects. Under quantum resonance conditions, the non-Hermitian-driven potential induces dynamics resembling a classical random walk, characterized by a linear increase in variance and a Gaussian momentum distribution. Notably, the growth rate of the variance diverges as the non-Hermitian parameter approaches zero. We also investigate the effects of deviations from the quantum resonance condition, referred to as the quantum non-resonance case, on the dynamics of quantum walk. Our findings reveal that in the Hermitian case, the quadratic growth of the variance is suppressed by these deviations, leading to the dynamical localization [39,40,41,42] of the quantum walk. In the non-Hermitian case, the classical random walk dynamics are also frozen under non-resonance conditions. Interestingly, the non-Hermitian driven potential enhances the saturation value of the linear entropy of internal degrees of freedom, indicating increased quantum entanglement. In contrast, deviations from the quantum resonance condition reduce the saturation value of the linear entropy, demonstrating the suppression of quantum entanglement as the system approaches the quantum non-resonance regime.

This paper is organized as follows. In Section 2, we describe our model. In Section 3, we show the classical random walk induced by the non-Hermitian driven potential. Section 4 involves the quantum walk in the quantum non-resonance condition. In Section 5, we discuss the quantum entanglement. Conclusions and discussions are presented in Section 6.

## 2. Realization of Quantum Walk with Spinor Kicked Rotor Model

The dimensionless Hamiltonian of the non-Hermitian spinor kicked rotor model reads

(1) $$H=\frac{p^{2}}{2}I+\left(K+i\lambda \right)\cos\left(\theta \right)\sigma_{z}\delta \left(t-t_{n}\right)\;,$$

where $p=-i\hbar_{\mathrm{eff}}\partial /\partial \theta$ is the angular momentum operator and is the angle coordinate, satisfying the commutation relation $\left[\theta ,p\right]=i\hbar_{\mathrm{eff}}$ with $\hbar_{\mathrm{eff}}$ as the effective Planck constant. The parameters *K* and λ control the strength of the real and imaginary parts of the kicking potential, respectively. The time $t_{n}$ counts the number of kicks and is therefore an integer, i.e., $t_{n}=1,2\ldots$. Here, $\sigma_{z}$ denotes the Pauli matrix, while I represents the identity matrix.

The two internal hyperfine states act as pseudospin degrees of freedom with S=±1/2 [18]. The coin operator realizing the superposition of two spin states takes the form

(2) $$M=\frac{1}{\sqrt{2}}\left(\begin{array}{cc} 1 & i\\ i & 1 \end{array}\right)\;,$$

which can be implemented with resonant microwave radiation [19,43]. In quantum walks, the momentum shift Δp is determined by the spin state: the momentum increases by one (Δp=1) for the spin state |↑〉 and decreases by one (Δp=−1) for the spin state |↓〉. Therefore, the shift operator is expressed as

(3) T=exp(inθ)|↑〉〈↑|+exp(−inθ)|↓〉〈↓|,

with n=1. For the kicked rotor model, the directed walk in momentum space can be realized by the quantum ratchet mechanism in quantum resonance condition, i.e., $\hbar_{\mathrm{eff}}=4\pi$. The momentum shift of Δp=±1 corresponds to nearest-neighbor coupling in momentum space, a condition that is approximately satisfied when $K/\hbar_{\mathrm{eff}}≳1$. Therefore, we set $K/\hbar_{\mathrm{eff}}=1.5$ in our investigation [19].

In the Hermitian case, the accuracy of the approximation $K/\hbar_{\mathrm{eff}}=1.5$ is justified by the expansion of the kicking evolution operator in the eigenbasis of the angular momentum operators, where the coefficient for nearest-neighbor coupling is dominant. In the non-Hermitian regime, this approximation remains valid because the non-Hermitian term primarily contributes to the growth of the norm of the time-evolved quantum state, a feature that has been accounted for in our definition of the variance. Specifically, at each kick, the non-Hermitian component of the kicking evolution operator, i.e., $\exp\left[\lambda \cos\left(\theta \right)\sigma_{z}/\hbar_{\mathrm{eff}}\right]$, induces norm growth, a hallmark of non-unitary evolution. Note that we conduct periodic renormalization for the time evolution of quantum state to compensate the effects of the divergent norm. As a result, the correspondence between the non-Hermitian kicking potential and the random walk holds true. Additionally, the non-Hermitian kicking potential does not affect the shift operator in Equation (3). We would like to emphasize that while the growth of the norm reflects changes in the overall amplitude of the wave function, the momentum variance is influenced by the non-Hermitian term’s effect on the system’s dynamics and quantum coherence. These two effects are complementary and do not contradict each other.

The time evolution of random walks in the quantum kicked rotor model involves a coin toss that induces a superposition of two internal states, followed by a kick evolution that causes a momentum shift of Δp=±1 for the walker in momentum space. This process establishes quantum entanglement between the external degrees of freedom (momentum) and the internal spin states, which is a feature of the quantum walk and distinguishes it from quantum state diffusion [19].

The eigenequation of the angular momentum operator is $p|\varphi_{n}\rangle =p_{n}|\varphi_{n}\rangle$, where the eigenvalue is $p_{n}=n\hbar_{\mathrm{eff}}$. The corresponding eigenstate in the position representation is given by $\langle \theta |\varphi_{n}\rangle =e^{in\theta }/\sqrt{2\pi }$. An arbitrary state $|\varphi_{n}\rangle$ can be expanded using the complete basis formed by $|\varphi_{n}\rangle$ as $|\psi \rangle =\sum_{n}\psi_{n}|\varphi_{n}\rangle$, where $\psi_{n}$ represents the expansion coefficients. The evolution of the quantum state over one period, from $t_{n}$ to $t_{n+1}$, is governed by $|\psi \left(t_{n+1}\right)\rangle =U|\psi \left(t_{n}\right)\rangle$, where the Floquet operator $U=U_{f}U_{K}$ consists of the free evolution operator $U_{f}=\exp\left(-ip^{2}I/2\hbar_{\mathrm{eff}}\right)$ and the kicking evolution operator $U_{K}=\exp\left[-i\left(K+i\lambda \right)\cos\left(\theta \right)\sigma_{z}/\hbar_{\mathrm{eff}}\right]$. It is important to note that in this context, the letter *U* does not indicate unitarity. In the quantum resonance condition (i.e., $\hbar_{\mathrm{eff}}=4\pi$), the $U_{f}$ is unity as $U_{f}\left(p_{n}\right)=\exp\left(-in^{2}2\pi \right)=1$, which has no effect on time evolution of quantum state. Therefore, the kicking evolution that induces the nearest-neighbor coupling of momentum sites acts as the shift operator. One period of the quantum walk is implemented by applying the shift operator $U_{K}$, followed by the coin operator, on a quantum state, namely $|\psi \left(t_{n+1}\right)\rangle =MU_{K}|\psi \left(t_{n}\right)\rangle$. To realize the directed walk, the initial state of the external degree of freedom must exhibit asymmetry with respect to the kicking potential. Accordingly, we choose the initial state as $|\psi \left(t_{0}\right)\rangle =\left(|\varphi_{0}\rangle +e^{i\phi }|\varphi_{1}\rangle \right)/\sqrt{2\pi }$, where ϕ=−π/2 [43].

## 3. Classical Random Walk Induced by Non-Hermiticity

**Figure 1:**
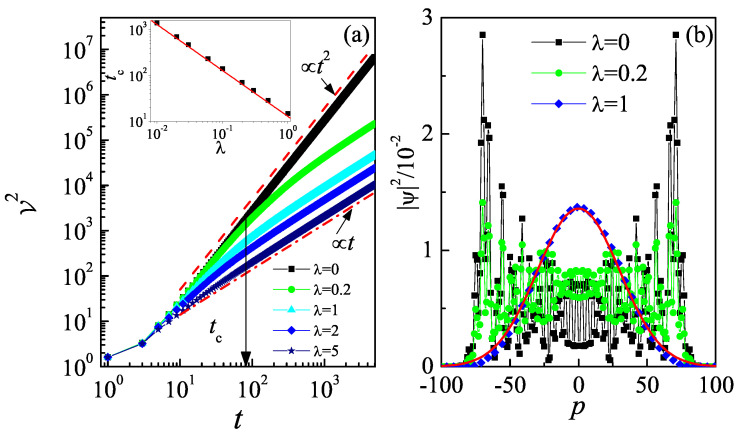
(**a**) The $V^{2}$ versus time for $\hbar_{\mathrm{eff}}=4\pi$ with λ=0 (squares), 0.2 (circles), 1 (triangles), 2 (diamonds), and 5 (pentagrams). The arrow marks the critical time $t_{c}$. The red dashed line and red dash-dotted line indicate the fitting functions $V^{2}\propto t^{2}$ and $V^{2}\propto t$, respectively. Inset: Dependence of $t_{c}$ on λ. The red line indicates the fitting function $t_{c}\propto \lambda^{-1}$. (**b**) Momentum distributions at the time t=100 for λ=0 (squares), 0.2 (circles), and 1 (diamonds). The red solid line indicates the Gaussian function ${\left|\psi \left(p\right)\right|}^{2}\propto e^{-p^{2}/\sigma }$ with σ≈1800.

We numerically investigate the time evolution of the momentum variance, $V^{2}=\langle p^{2}\rangle /N-{\left(\langle p\rangle /N\right)}^{2}$, for different values of λ. Here, 〈·〉=〈ψ(t)|·|ψ(t)〉 denotes taking the expected value with respect to the pure state, and N=〈ψ(t)|ψ(t)〉 represents the norm of the time-evolved quantum state. This definition of the mean value of observables eliminates the contribution of the norm, which typically exhibits exponential growth over time in non-Hermitian systems. In the Hermitian case (λ=0), the $V^{2}$ grows quadratically with time, indicating a clear emergence of quantum walk ( see Figure 1a). In this situation, the momentum distribution exhibits two prominent peaks at $p_{\pm }\left(t\right)=p_{0}\pm p_{max}\left(t\right)$ [43], where the initial momentum $p_{0}=0.5$. The linear increase in $p_{max}$ over time results in the quadratic growth of $V^{2}$. For a small value of λ (e.g., λ=0.2), $V^{2}$ initially follows the quadratic growth observed in the Hermitian case λ=0 within a finite time interval $\left(t<t_{c}\right)$ and transitions to linear growth, i.e., $V^{2}=Gt$, after a long time evolution. For $\left(t<t_{c}\right)$, the momentum distribution also resembles that of a quantum walk (see Figure 1b). We further numerically investigate the $t_{c}$ for different λ. Our result demonstrates that the $t_{c}$ decreases inversely with the increase in λ (see the inset in Figure 1a). Note that the linear growth of $V^{2}$ is widely recognized as an indicator of classical random walks [44]. To confirm this, we numerically investigate the probability density distribution in momentum space for $t>t_{c}$. Our results reveal a Gaussian distribution, i.e., ${\left|\psi \left(p\right)\right|}^{2}\propto e^{-p^{2}/\sigma }$, which demonstrates the emergence of classical random walk behavior induced by the non-Hermitian driven potential (see Figure 1b for λ=1).

It is known that the dissipation effect in open systems can be treated in some approaches as a manifestation of non-Hermitian dynamics. One dominating approach involves microscopic models where the system is coupled to a heat bath. By tracing out the bath’s degrees of freedom, a statistical description can be obtained in terms of a reduced density operator, leading to a non-unitary time evolution that cannot, however, be directly associated with any non-Hermitian Hamiltonian. Open systems with particle loss and creation serve as paradigm cases of non-Hermitian quantum mechanics.

The kicking evolution operator $U_{K}$ can be expanded as $U_{K}=\sum_{n,r=-\infty }^{\infty }{\left(-i\right)}^{r}I_{n-r}\left(\lambda /\hbar_{\mathrm{eff}}\right)$ $J_{r}\left(K/\hbar_{\mathrm{eff}}\right)e^{in\theta }$, where $I_{n}$ is the modified Bessel function of order *n*, and $J_{r}$ is the Bessel function of the first kind of order *r* [45,46]. Based on this expansion, one can obtain the matrix elements of the $U_{K}$ in the angular momentum representation, i.e., $\langle m|U_{K}|n\rangle =\sum_{r=-\infty }^{\infty }{\left(-i\right)}^{r}I_{m-n-r}\left(\lambda /\hbar_{\mathrm{eff}}\right)J_{r}\left(K/\hbar_{\mathrm{eff}}\right)$. In the limit λ→0, the matrix degenerates to the Hermitian case, i.e., $\langle m|U_{K}|n\rangle ={\left(-i\right)}^{m-n}J_{m-n}\left(K/\hbar_{\mathrm{eff}}\right)$, since $I_{n}\left(0\right)=1$ when n=0 and $I_{n}\left(0\right)=0$ otherwise. The Bessel function has a key feature $J_{n}\left(x\right)∼0$ when n≫x, meaning it decays rapidly to zero for large *n* [39,40]. Therefore, in the Hermitian case, the $U_{K}$ leads to nearest-neighbor coupling in angular momentum space when $K/\hbar_{\mathrm{eff}}≃1$ [19]. In contrast, for a specific λ>0, the $U_{K}$ can induce long-range coupling as the modified Bessel function $I_{n}\left(\lambda \right)$ increases continuously with λ. We have found that the non-Hermitian effects, represented by such long-range coupling, can significantly influence quantum dynamics, including quantum diffusion, directed current, and quantum scrambling, in Floquet systems [45,46]. Consequently, it is reasonable to believe that such long-range coupling results in the emergence of the classical random walk. Our finding of classical random walk behavior in the quantum regime highlights the quantum-to-classical transition in the quantum kicked rotor model [47,48,49,50,51].

**Figure 2:**
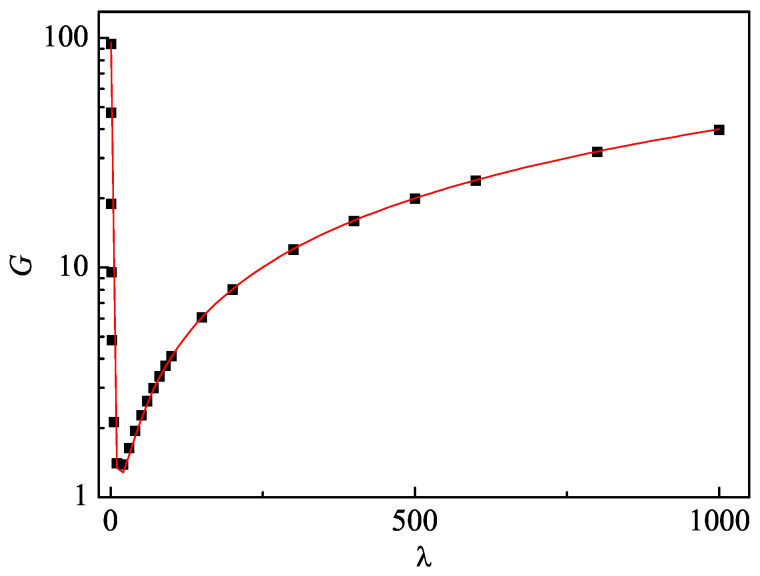
The *G* versus λ. The red solid line indicates the fitting function of the form $G\approx \alpha (\beta +\lambda^{2})/\lambda$ with α≈0.04 and β≈238.1. The parameters are the same as in Figure 1.

We numerically investigate the growth rate $G=dV^{2}/dt$ of the linear growth of the variance for various λ. Interestingly, the *G* decreases initially with the increase in λ when λ is smaller than a critical value, after which the *G* increases unboundedly with λ (see Figure 2). The fitting function of the numerical results takes the form $G\approx \alpha (\beta +\lambda^{2})/\lambda$ with α≈0.04 and β≈238.1, revealing the divergence of the *G* with λ→0 [45].

## 4. Dynamical Localization of Quantum Walk in Quantum Non-Resonance Condition

**Figure 3:**
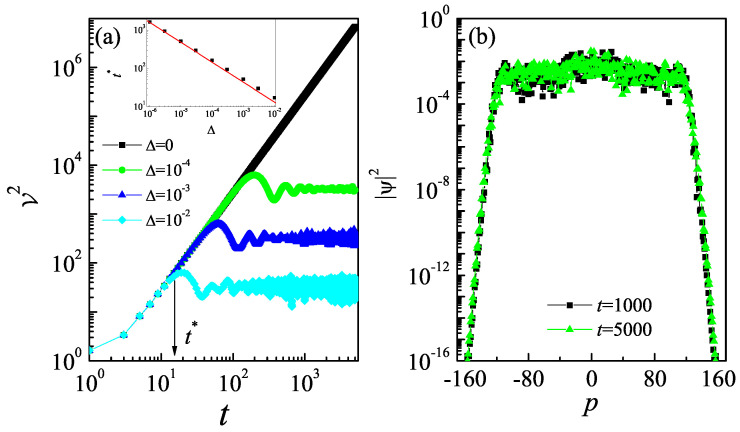
(**a**) The $V^{2}$ versus time for $\hbar_{\mathrm{eff}}=4\pi +\Delta$ with Δ=0 (squares), $10^{-4}$ (circles), $10^{-3}$ (triangles), and $10^{-2}$ (diamonds). The arrow marks the critical time $t^{*}$. Inset: Dependence of $t^{*}$ on Δ. The red line indicates the fitting function $t^{*}\propto \Delta^{-0.52}$. (**b**) Momentum distributions for $\Delta =10^{-4}$ with t=1000 (squares) and 5000 (triangles). The value of the non-Hermitian parameter is λ=0.

Note that the quantum resonance condition is an exceptional case, as it will be hit with zero probability if the parameter $\hbar_{\mathrm{eff}}$ is chosen at random. Any infinitesimal deviation from the quantum resonance values leads to the more generic case of the quantum non-resonance condition. In such cases, the initial time evolution of observables resembles the behavior at the quantum resonance condition, indicating a transient effect. It is well established that the Hermitian kicked rotor model exhibits rich physical phenomena, such as ergodicity breaking and dynamical localization, in the quantum non-resonance regime [52,53,54,55,56]. Motivated by this, we further investigate the dynamics of quantum walks beyond the quantum resonance condition, i.e., $\hbar_{\mathrm{eff}}=4\pi +\Delta$. For a very small Δ (e.g., $\Delta =10^{-4}$ in Figure 3a), the $V^{2}$ follows that of Δ=0 during finite time evolution, i.e., $t<t^{*}$, and gradually saturates when $t>t^{*}$, revealing the dynamical localization of the quantum walk. Moreover, both the critical time $t^{*}$ and the saturation level decrease with the increase in Δ. It is reasonable to believe that the quantum interference effects, which lead to dynamical localization, suppress the quantum walk in momentum space. The critical time $t^{*}$ is in the power-law function of Δ, i.e., $t^{*}\propto \Delta^{-0.52}$ (see the inset in Figure 3a). The localization of the quantum walk is also confirmed by the probability density distribution in momentum space. As shown in Figure 3b, our results reveal that the momentum distributions at different times almost overlap and remain unchanged as time evolves.

**Figure 4:**
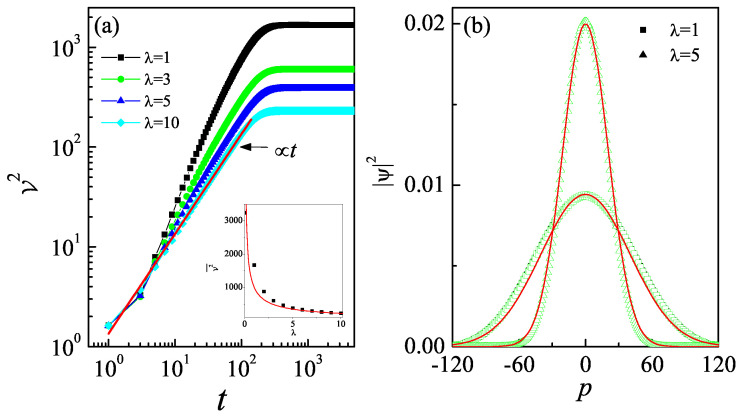
(**a**) The $V^{2}$ versus time for $\Delta =10^{-4}$ with λ=1 (squares), 3 (circles), 5 (triangles), and 10 (diamonds). The red solid line indicates the fitting function $V^{2}\propto t$. Inset: The time-averaged value of variance $\bar{V^{2}}$ versus λ. The red line indicates the fitting function $\bar{V^{2}}\propto \lambda^{-0.65}$. (**b**) Momentum distributions at time t=1000 (solid symbols) and 2000 (empty symbols) with λ=1 (squares) and 5 (triangles). Red solid lines indicate the Gaussian function ${\left|\psi \left(p\right)\right|}^{2}\propto e^{-p^{2}/\sigma }$ with σ≈3250 and 850 for λ=1 and 5, respectively.

We further investigate the non-Hermitian effects on quantum walk under quantum non-resonance conditions. Our results show that for a specific λ value (e.g., λ=1 in Figure 4a), the $V^{2}$ increases linearly over a finite time interval and then saturates after a long time evolution. This clearly reveals the dynamical localization of the classical random walk due to the non-Hermitian driven potential. Moreover, the saturation level of $V^{2}$ decreases with the increase in λ. We use the time-averaged values, i.e., $\bar{V^{2}}=\sum_{j=1}^{N}V^{2}\left(t_{j}\right)/N$, to quantify the saturated variance values, where *N* is the total number of kicking periods. Numerical simulations indicate that for relatively large λ (e.g., λ=1 ), a choice of N=5000 kick periods ensures a good approximation of the saturated values through $\bar{V^{2}}$. Our result demonstrates that $\bar{V^{2}}$ decreases with increasing λ, which can be fitted as the power-law function $\bar{V^{2}}\propto \lambda^{-0.65}$ (see the inset in Figure 4a). We also numerically investigate the momentum distributions when the dynamical localization of a classical random walk occurs. Our results demonstrate that for a specific value of λ (e.g., λ=1), the momentum distributions are in the shape of Gaussian wavepackets ${\left|\psi \left(p\right)\right|}^{2}\propto e^{-p^{2}/\sigma }$, with a constant width σ as time evolves, corresponding to the saturation of the variance $\bar{V^{2}}$. Additionally, the width σ decreases with the increase in λ, leading to a reduction in $\bar{V^{2}}$.

## 5. Quantum Entanglement

**Figure 5:**
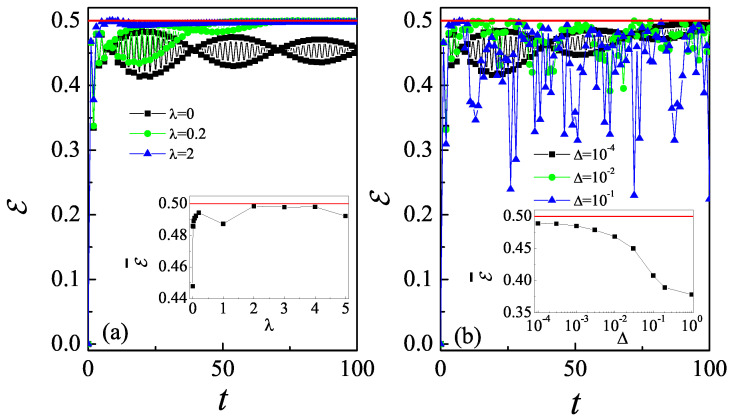
(**a**) Time dependence of linear entropy E for $\hbar_{\mathrm{eff}}=4\pi$ with λ=0 (squares), 0.2 (circles), and 2 (triangles). Inset: The time-averaged value of linear entropy $\bar{E}$ versus λ. (**b**) Linear entropy E versus time for $\hbar_{\mathrm{eff}}=4\pi +\Delta$ with $\Delta =10^{-4}$ (squares), $10^{-2}$ (circles), and $10^{-1}$ (triangles). Inset: The $\bar{E}$ versus Δ. Red lines in the main plot (insets) indicate the value E=0.5 ($\bar{E}=0.5$). The non-Hermitian parameter is λ=0.

QRWs inherently differ from CRWs in that QRWs can naturally generate entanglement between spatial degrees of freedom and internal states [2,4,5], enabling potential applications in quantum computation and quantum information [3,7]. We numerically investigate quantum entanglement between internal degrees of freedom, quantified by the linear entropy $E=1-\mathrm{Tr}(\rho_{s}^{2})$. Here, $\rho_{s}$ is the reduced density matrix of the spin, obtained by tracing out the external degrees of freedom from the system’s density matrix [57,58]. Our results show that under the quantum resonance condition ($\hbar_{\mathrm{eff}}=4\pi$), the linear entropy *S* increases rapidly and then exhibits periodic oscillations around E≈0.45 over time when the system is Hermitian (see λ=0 in Figure 5a), indicating the emergence of quantum entanglement. For relatively small λ (e.g., λ=0.2), E initially follows the behavior of the Hermitian case for a finite time before approaching saturation as time evolves. For much larger λ, E saturates rapidly with time. Furthermore, we numerically calculate the time-averaged linear entropy, $\bar{E}=\left(1/t_{M}\right)\sum_{n=1}^{M}E\left(t_{n}\right)$, to quantify its saturation value. Our results show that $\bar{E}$ initially increases rapidly before saturating at approximately $\bar{E}\approx 0.5$ as λ increases (see the inset in Figure 5a), indicating the enhancement of entanglement facilitated by the non-Hermitian-driven potential.

We also investigate the effects of the deviations from the quantum resonance condition, $\hbar_{\mathrm{eff}}=4\pi +\Delta$, on entanglement. For very small deviations (e.g., $\Delta =10^{-4}$ in Figure 5b), the E exhibits periodic oscillations over a finite time interval, which gradually vanish. For intermediate deviations (e.g., $\Delta =10^{-2}$), E fluctuates around a saturation value, with the magnitude of fluctuations increasing as Δ grows. The time-averaged value of linear entropy decreases with the increase in Δ (see the inset in Figure 5b), demonstrating the suppression of entanglement as the system approaches the quantum non-resonance regime. Our results shed light on manipulating quantum entanglement via the QRW model.

## 6. Conclusions and Discussions

Non-Hermitian systems serve as a versatile platform for exploring fundamental concepts [59], including new topological phases [60,61], quantum information scrambling [46,62,63,64], and quantum chaos [65]. In this work, we numerically investigate the quantum walk dynamics in the momentum space of the spinor kicked rotor model with a non-Hermitian-driven potential. For $\hbar_{\mathrm{eff}}=4\pi$, the variance $V^{2}$ in momentum space grows linearly with time when the non-Hermitian parameter λ is sufficiently large. The momentum distributions exhibit Gaussian wavepacket shapes, signifying the emergence of classical random walk behavior. Remarkably, the growth rate *G* of the variance diverges as λ→0. For $\hbar_{\mathrm{eff}}=4\pi +\Delta$, dynamical localization of the quantum walk is observed over time. This phenomenon manifests as the saturation of both quadratic growth (λ=0) and linear growth (λ≠0) of the variance. We find that the linear entropy E increases rapidly and eventually saturates as time evolves. The saturation value $\bar{E}$ increases with increasing λ, stabilizing at approximately 0.5 for sufficiently large λ, which demonstrates that the non-Hermitian-driven potential effectively enhances quantum entanglement. Interestingly, for a specific λ, the $\bar{E}$ decreases with the increase in Δ, revealing the suppression of quantum entanglement by the quantum non-resonance condition.

Our results offer practical strategies for optimizing entanglement generation and control in quantum systems, by demonstrating how parameters such as the non-Hermitian potential and quantum resonance deviations influence entanglement dynamics. The linear growth in the variance of the momentum distribution with increasing non-Hermitian parameter indicates a quantum-to-classical transition in the quantum resonance case [19]. Additionally, the dynamical localization of the quantum walks in both Hermitian and non-Hermitian regimes, caused by the deviation from the quantum resonance condition, sheds light on quantum transport in disordered systems. 

## Data Availability

The original contributions presented in this study are included in the article. Further inquiries can be directed to the corresponding author.
